# Migraine triggered seizures and epilepsy triggered headache and migraine attacks: a need for re-assessment

**DOI:** 10.1007/s10194-011-0344-2

**Published:** 2011-04-24

**Authors:** Paul T. G. Davies, C. P. Panayiotopoulos

**Affiliations:** Neurosciences, John Radcliffe Hospital, Oxford, UK

In this issue of the Journal, Belcastro and associates review terminology and classification issues for migralepsy, hemicrania epileptica, post-ictal and ictal headache [1]. They raise key points such as ictal headache and visual seizures are often misdiagnosed as migraine, “migralepsy” is unlikely to exist and an “epilepsy-migraine sequence” is much more common and well documented than the dominant view of a “migraine-epilepsy sequence”. Their relevant proposals need appropriate attention by the committee of the international classification of headache disorders (ICHD) as well as the physicians in their clinical practice because of the consequences that misdiagnosis may have on patients.

Misdiagnosis between epilepsy and migraine is considerable though their differentiation should not be difficult on clinical grounds [2]. The problem is that emphasis is unduly placed on individual symptoms, rather than on a comprehensive synthetic analysis of their quality, chronological sequence and other clustering features. There are many examples of such errors where visual seizures are unquestionably diagnosed as acephalgic migraine and more often migraine with aura (if seizures are followed by post-ictal headache) or basilar migraine with occipital paroxysms (which does not exist, because this is a syndrome of idiopathic childhood occipital epilepsy). Elementary visual hallucinations of occipital seizures develop rapidly within seconds, are brief in duration (2–3 min), are usually coloured and circular. These are fundamentally different from the visual aura of migraine, which develops slowly in minutes, is longer lasting (≥5 min) and mainly achromatic with linear patterns [3].

## Migralepsy

According to the ICHD-II 1.5.5, “migraine-triggered seizure (sometimes referred to as migralepsy)” denotes an epileptic seizure that occurs “during or within one hour after a migraine aura” [3]. However, the evidence of this “migraine-seizure” sequence is weak and the proposed criterion of 1 h gap between the end of the “aura” and the start of an epileptic seizure is entirely arbitrary

Migralepsy is an old term derived from migra(ine) and (epi)lepsy, coined by Dr Douglas Davidson, but mainly attributed to Lennox and Lennox, which we quote, “a given seizure may be a composite of symptoms encountered in epilepsy and migraine. Such cases are more common in children than in adults. In our experience, the migraine-like symptoms appear first−the characteristics of ophthalmic migraine with perhaps nausea and vomiting, followed by symptoms−characteristic of epilepsy, impairment or loss of consciousness and involuntary muscle movement. Dr Douglas Davidson calls such hybrid phenomena as ‘migralepsy’” [4].

There should be no reason why epileptic seizures, which are so vulnerable to precipitating factors, could not be susceptible to cortical changes induced by migraine [2, 3]. However, by probability of prevalence these cases should have symptoms of typical visual aura of migraine followed by epileptic seizures. Contrary to this, reviews of the approximately 50 reported cases of “migralepsy” showed that most of them, including two of the three illustrative cases of Lennox and Lennox, had symptoms of visual seizures as defined above, which were interpreted as migraine aura [2, 3, 5]. Thus, it is probable that these are genuine occipital seizures imitating migraine. There are also some cases with atypical presentations or bizarre symptomatology that could be diagnosed either as migraine or epilepsy.

## Ictal headache, hemicrania epileptica and status epilepticus migrainosus

Headache as a symptom of an epileptic seizure is well documented in many publications. However, this ictal headache manifests with multiple and diverse symptomatology, such as a sensation of bifrontal pressure, vague ache in the head, sharp stabbing retro-orbital pains or a sensation of electricity passing through the head of varying intensity, discomfort and localisation. These are often cephalic sensations with or without true ache and may occur at the onset or in the progression of a seizure. As a rule, ictal headache is associated with other seizure symptomatology emanating from the affected epileptogenic zone. It may be exceptional to have headache as a sole manifestation of a seizure and this is more unlikely to be consistent in all seizures of the same patient. Ictal headache is unlikely to be confused with migraine though this may be the case in some exceptional patients and circumstances referred to as hemicrania epileptica and status epilepticus migrainosus

The ICHD-II includes ictal headache amongst a broad group of “headache attributed to epileptic seizure” (7.6), which may be ictal, periictal, postictal, interictal or comorbid, without specifying characteristics of these headaches [3]. Of ictal headaches, the ICHD-II describes only hemicrania epileptica (7.6.1) though this is extremely rare and if it exists is unlikely to fulfil the proposed diagnostic criteria of A. Headache lasting seconds to minutes, with features of migraine, fulfiling criteria C and D, B. The patient is having a partial epileptic seizure, C. Headache develops synchronously with the seizure and is ipsilateral to the ictal discharge and D. Headache resolves immediately after the seizure. Status epilepticus migrainosus has been described in 4–5 cases, of mainly occipital lobe epilepsy and its epileptic nature is documented with ictal EEG [1, 5]. Thus, this is also an ictal headache imitating migraine.

## Postictal headache and the epilepsy-migraine sequence

Postictal headache occurs in 10–40% of patients with partial or generalised seizures and may be indistinguishable from migraine particularly if associated with vomiting and photophobia (ICHD-II 7.6.2) [3]. Another similarity with migraine is that postictal headache often occur 3–15 min from a definite epileptic seizure [2, 3], a situation described by Blau in migraine as the “asymptomatic interval” between the end of migraine aura and the onset of headache [6].

Postictal headache is particularly common in occipital epilepsy occurring in more than half of patients even after brief simple visual seizures [2, 3]. This high prevalence of postictal headache after occipital seizures of any cause may indicate that the occipital lobes are preferentially associated with trigeminovascular or brain stem mechanisms responsible for migraine headache [2, 3].

Based on these findings, it is reasonable to propose that occipital seizures often generate a true migraine attack. This “occipital seizure-migraine” sequence appears to be much commoner than the previously prevailing view of migraine visual aura triggering epileptic seizures [2, 3].

## Conclusions

In view of the above uncertainties and the high prevalence of misdiagnosis, there is a need for collaboration between the relevant headache and the epilepsy classification committees to critically re-assess these matters, which are of significant diagnostic, management and pathophysiological importance. Achieving clarity and consensus in this area may be challenging but with the ICHD-III on the horizon, now is the time for this to happen.
